# Identification of the cDNA Encoding the Growth Hormone Receptor (*GHR*) and the Regulation of *GHR* and *IGF-I* Gene Expression by Nutritional Status in Reeves’ Turtle (*Chinemys reevesii*)

**DOI:** 10.3389/fgene.2020.00587

**Published:** 2020-06-09

**Authors:** Wenlu Zhu, Yuhui He, Zhuohao Ruan, Xiquan Zhang, Liangyuan Liao, Yicong Gao, Nani Lin, Xiancan Chen, Rui Liang, Wen-sheng Liu

**Affiliations:** ^1^College of Marine Sciences, South China Agricultural University, Guangzhou, China; ^2^Guangdong Province Key Laboratory for Animal Genomics and Molecular Breeding, South China Agricultural University, Guangzhou, China; ^3^Guangdong Province Engineering Research Centre of Aquatic Immunization and Aquaculture Health Techniques, South China Agricultural University, Guangzhou, China

**Keywords:** *Chinemys reevesii*, Reeves’ turtle, GHR, IGF-I, starvation

## Abstract

*Chinemys reevesii* (Reeves’ turtle) is a slow-growing reptile that is distributed widely across China. Prior to this study, the cDNA sequence of the growth hormone receptor (*GHR*) in the Reeve’s turtle, or how periods of starvation might influence the gene expression of *GHR* and insulin-like growth factor I (*IGF-I*) in this species, were unknown. Here, we identified the full-length sequence of the cDNA encoding GHR in Reeves’ turtle by using RT-PCR and RACE. The full-length *GHR* cDNA was identified to be 3936 base-pairs in length, with a 1848 base-pair open reading frame (ORF) that encodes a 615 amino acid protein. Analysis showed that *GHR* mRNA was detectable in a wide range of tissues; the highest and lowest levels of expression were detected in the liver and the gonad, respectively. *IGF-I* was also expressed in a range of tissues, but not in the gonad; the highest levels of *IGF-I* expression were detected in the liver. After 4 weeks of fasting, the expression levels of *GHR* and *IGF-I* in the liver had decreased significantly; however, these gradually returned to normal after refeeding. We report the first cloned cDNA sequence for the *GHR* gene in the Reeve’s turtle. Our findings provide a foundation from which to investigate the specific function of the GHR in Reeve’s turtle, and serve as a reference for studying the effects of different nutrient levels on *GHR* expression in this species.

## Introduction

Growth hormone (GH) is a core hormone in the biological axis that controls growth and development, and is specifically responsible for promoting growth. In order to exert its physiological function, GH first binds to GH receptors (GHRs) on the surface of the target cell membrane. GHRs mediate the passage of an extracellular signal into the cell in order to produce a series of physiological effects, thus promoting growth and development. The GH/insulin-like growth factor (IGF) axis is a vital endocrine system in the regulation of vertebrate growth (Reindl and Sheridan, 2012). The individual components of the GH/IGF axis, including GH, IGF, and their receptors and binding proteins, not only interact to coordinate growth, they also coordinate a range of other biological processes, including metabolism, gonadal development, osmoregulation, social behavior, and immunity (Bergan-Roller and Sheridan, 2018). Generally, the physiological activity of GH is triggered when it binds to the growth hormone receptor (GHR) in target tissues and thus induces the production of IGF-I; in turn, the ability of GH to promote growth is mediated by IGF-I (Kopchick and Andry, 2000).

In animals, nutritional availability can exert profound influence on growth and the activity of the GH/IGF system (Ross, 2000; Gabillard et al., 2006; Reindl and Sheridan, 2012). A considerable volume of research has explored the expression of *GH* and *IGF* genes and their relative response to nutritional status. For example, one study reported that the levels of *IGF-I* and *GHR* in the liver of young growing rats declined when fasting or when maintained on a protein-deficient diet (Straus and Takemoto, 1990). In another study, 2 weeks of fasting resulted in a significant reduction of *IGF-I* mRNA in the liver and plasma of both male and female tilapia (Uchida et al., 2003). mRNA levels of *IGF-I* and *GHR* were also significantly lower in a fasted group of masu salmon compared with those in the group that was fed (Fukada et al., 2004; Kawaguchi et al., 2013; Price et al., 2013); similar results have been reported for the catfish (Small et al., 2006; Peterson and Waldbieser, 2009). Other research studies have reported that food restriction significantly increased *GH* but reduced the mRNA levels of *IGF-I* in grouper (Pedroso et al., 2006), rabbitfish (Ayson et al., 2007), catfish (Small and Peterson, 2005), tilapia (Fox et al., 2009), and Atlantic salmon (Wilkinson et al., 2006). The expression of *GHR* mRNA was significantly reduced in diploid and triploid crucian carp after just 1 week of starvation (Zhong et al., 2012). However, fasting had no effect on the expression of *GHR* mRNA in the liver of tilapia (Fox et al., 2006).

Most studies related to nutritional status and the GH/IGF axis have been carried out in mammals or fish; very little is known about this axis in Testudines. Reeves’ turtle is a fresh water species that is widely distributed from Japan to southern China across eastern Asia (Ly et al., 2013). This species has significant economic value as an aquatic food source, as a pet, and as a source of traditional medicine. Consequently, efforts to cultivate this species in China have increased significantly over recent years. Reeves’ turtle is generally a relatively slow-growing and long-lived species. During the breeding process, the Reeves’ turtle often undertakes periods of intermittent, prolonged, or complete food deprivation; the factors underlying such behavior include high stocking densities, changes in temperature, seasonal fluctuations, and other environmental factors. When food is scarce, this species can still survive for a long time period. Over recent years, research studies have investigated how nutritional status in the Reeve’s turtle relates to compensatory growth (Wang et al., 2011; Xu et al., 2014), metabolic rate (Belkin, 1965; Sievert et al., 1988; Colman et al., 2014), and physiological and biochemical responses. Some researchers have specifically investigated the effects of fasting on tissue glycogen content (Partata and Marques, 1994; Moon et al., 1999), the serum concentration of triglycerides and β-hydroxybutyrate (Price et al., 2013), and enzymic activity (Penney and Papademas, 1975). However, beyond this, little is known about the impact of nutritional status on the molecular mechanisms that regulate the growth of the Reeve’s turtle.

In a previous study, we demonstrated that the Reeves’ turtle exhibits a higher nucleoplasmic ratio and fewer secretory granules in pituitary GH cells than other animal species (Liu and Li, 2005). Moreover, we found that intron 1 of the *GH* gene in Reeves’ turtle was much longer than that in other species, and that this feature might correlate with specific patterns of gene expression (Liu W.S. et al., 2016). Thus, it is evident that GH exhibits unique histological and genetic features in Reeves’ turtle. However, the question of whether Reeve’s turtle, and other animal species, utilize different coping strategies when faced with food deprivation, particularly with regards to gene expression in the GH/IGF axis, has yet to be answered, and deserves particular attention.

In the present study, we cloned the cDNA sequence of *GHR* and *IGF-I* from samples of liver tissue from the Reeves’ turtle. We then analyzed the homology of the *GHR* cDNA from Reeves’ turtle and investigated the transcriptional changes in *GHR* and *IGF-I* in response to food deprivation. Our findings provide a significant advancement in our knowledge of the potential mechanisms involved in the ability of the Reeves’ turtle to endure food deprivation.

## Materials and Methods

### Animals and Sampling

Reeves’ turtles (body weight range: 75–150 g) were obtained from Guangzhou Chengchang Biotechnology Co., Ltd., and maintained in a plastic case (100 cm × 200 cm × 30 cm) containing filtered and aerated water at a constant temperature (25 ± 1°C). The turtles were fed twice daily at 9:00 am and 18:00 pm with a commercial diet (3% body weight) (Tianbang, Guangzhou, China).

After 2 weeks of acclimation, the turtles were randomly divided into two groups. In the experimental group, Reeve’s turtles were starved for 28 days and then re-fed twice a day for 28 days. At the end of the experiment, three male and three female turtles from each group were anesthetized with tricaine methanesulfonate (MS-222, 200 mg/L) to perform analyses in order to detect the effect of starvation on gene expression. Various tissues (brain, liver, adipose, kidney, spleen, muscle, foregut, midgut, hindgut, stomach, bladder, lung, heart, and testis/ovary) were frozen immediately in liquid nitrogen upon surgical resection to avoid degradation, then stored at −80°C prior to RNA isolation. In addition, we collected liver samples from turtles (*n* = 3) in the experimental group on days 0, 1, 7, 14, 21, and 28 days after fasting, and on days 1, 7, 14, 21, and 28 days after refeeding, in order to investigate the dynamic variation of gene expression during fasting and refeeding.

All samples were dissected, immediately frozen in liquid nitrogen, and then stored at −80°C to await total RNA isolation. All experimental procedures were consistent with the guidance and protocols approved by the Animal Care Committee of South China Agricultural University (Guangzhou, China) (Approval number: SCAU#0011, 3rd August 2010).

### RNA Isolation and Reverse Transcription

Total RNA was isolated with Trizol reagent (Takara Biochemicals, Kusatsu, Japan) in accordance with the manufacturer’s protocol. Following ethanol precipitation, the extracted RNA was re-suspended in 30–80 μl of RNase-free water. RNA integrity was subsequently evaluated by 1.5% agarose gel electrophoresis; RNA purity was evaluated by ultraviolet spectrophotometry at 260 and 280 nm. RNA samples exhibiting three RNA bands (28S, 18S, and 5S) and with an A260/A280 ratio of 1.8–2.2 were subsequently used for cDNA synthesis. Reverse transcription was carried out with 1μg of total RNA and Revert Aid^TM^ Reverse Transcriptase (Takara Biochemicals, Kusatsu, Japan), in accordance with the manufacturer’s protocol. The synthesized cDNAs were stored at −20°C and then used for cloning and mRNA quantification of target genes.

### Partial Cloning of *GHR*, *IGF1*, and the β*-Actin* Genes

First, we designed degenerate primers (Table 1) for *GHR* (GenBank Number: MH460865.1), *IGF1* (GenBank Number: XM_005303696.2), and β*-actin* (GenBank Number: MH460867.1). Primers were designed using Primer 5.0 software and were based on known sequences listed in the GenBank database. All primers were synthesized by Sangon (Shanghai, China). Polymerase chain reaction (PCR) was then used to amplify fragments of the *GHR*, *IGF1*, and β*-actin* genes. Amplification reactions were carried out on a BIO-RAD S1000 Thermal Cycler PCR (United States) using TransStart KD plus DNA polymerase (TransGen Biotech, Beijing, China). The PCR program featured an initial step of 95°C for 5 min followed by 35 cycles of denaturation (94°C for 30 s), annealing (at the designated temperature for each primer for 1 min) and extension (68°C for 1 min). After the 35 cycles, there was a final extension step of 68°C for 5 min; the reaction was terminated by incubation at 4°C. PCR products were separated by 1.5% agarose gel electrophoresis, purified, and then inserted into the pMD18T Simple vector (Takara Biochemicals, Kusatsu, Japan). The engineered construct was then transformed into *E. coli* DH5α competent cells (Takara Biochemicals, Kusatsu, Japan). This resulted in three positive clones for each PCR fragment. These fragments were sequenced by BGI Genomics (Shenzhen, China). The resulting sequences were then verified by using the Basic Local Alignment Search Tool (BLAST)^1^ on the National Center for Biotechnology Information (NCBI) database^2^.

**TABLE 1:** The primers used for gene cloning and RT-PCR in this study.

**Gene**	**Primers**	**Application**	**Sequence (5′–3′)**	**Tm (°C)**
*GHR*	*GHR*-F	Gene cloning	GACTGATCAATATCTGAGGTGC	52
	*GHR*-R		AATGAGACGAAATGGAGGG	
*IGF-I*	*IGF-I*-F	Gene cloning	ACAAGTAGAGGGAACACAGG	55
	*IGF-I* -R		GAAAGGGGTAGACAAACAAA	
β*-actin*	β*-actin-F*	Gene cloning	ACAGAAAGAGGCTACAGCTT CACC	54
	β*-actin-R*		GCTTTTCCTTGATGTCACGCA	
*GHR*	GSP1	5′RACE	TAGCCAGCCACGACAGCTACA AGCCACAGC	68
	NGSP1		GACAGACGGATTGGACACTGAC AGGCTCCTGGG	
*GHR*	GSP2	3′RACE	CCCAGGAGCCTGTCAGTGTCCA ATCCGTCTGTC	67
	NGSP2		GCTGTGGCTTGTAGCTGTCGT GGCTGGCTA	
*GHR*	*GHR*-F	RT-PCR	CTCCATCTTAGCCAGCCACG	60
	*GHR*-R		GTCCAGAATCATCATCTTTCACTCC	
*IGF-I*	*IGF-I*-F	RT-PCR	TCCTGTGCCCCTCAAAAGC	63
	*IGF-I*-R		AACAAAACGGAACCCCAACTC	
β*-actin*	β*-actin-F*	RT-PCR	GCTGGCCGTGATCTGACGGACTA	64
	β*-actin-R*		TGGAAGAGGGCTTCTGGGCACCT	

### RACE and Bioinformatic Analysis of the *GHR* Gene

Based on the partial cDNA sequence obtained by homology-based cloning, we designed gene specific primers (GSP) to amplify the 5′-end and 3′-end of *GHR* (Table 1). We then used Rapid Amplification of cDNA Ends (RACE) to acquire the full-length cDNA of *GHR* using the SMARTer RACE cDNA Amplification Kit (Clontech:634923) (Clontech, United States). In accordance with the manufacturer’s protocols, the first strand 3′-RACE-ready cDNA and 5′-RACE-ready cDNA were prepared and used as templates for the first round of PCR amplification. Products of the first PCR run were then diluted by a factor of 50 and used as templated for nested PCR amplification reactions. GSP1, NGSP1, GSP2, and NGSP2 primers were used for the 5′-end first round PCR, 5′-end nested PCR, 3′-end first round PCR, and 3′-end nested PCR, respectively. The two different PCR protocols were carried out under the same conditions, with a thermal cycling profile of 94°C for 5 min, 25 cycles at 94°C for 10 s, 68°C for 15 s, and 72°C for 3 min. The amplified RACE-PCR products were purified and cloned into pMD18T Simple vectors and sequenced on both strands, as described above.

By assembling the 5′-end, core fragment, and 3′-end, it was possible to deduce the full-length cDNA sequence of *GHR*. The deduced sequence was confirmed by using the BLAST algorithm on the NCBI database. The open reading frame (ORF) of *GHR* was predicted by ORF Finder tools^3^ and the deduced amino acid sequences were obtained using DNAMAN software. The signal peptide was predicted using SignalP 4.1 Server^4^. Glycosylation sites were predicted using the NetNGlyc 1.0 Server^5^. Transmembrane helices were predicted using the TMHMM Server version 2.0^6^. Multiple alignments of the *GHR* gene sequence were performed by ClustalW2^7^. Phylogenetic analysis was conducted using the neighbor-joining (NJ) method with 1000 bootstrap by Mega 6 software^8^.

### Real-Time Quantitative PCR

The primers used for real-time quantitative PCR are given in Table 1. The β*-actin* gene was amplified as an internal control (Liu W.S. et al., 2016). Real-time quantitative PCR was carried out with an ABI 7500 Fast Real-Time PCR System (ABI Applied Biosystems, Foster City, CA, United States). RT-PCR was performed in a total volume of 20 μl containing TransStart^®^ Tip Green qPCR SuperMix (TransGen Biotech, Beijing), 0.2 μM of each primer, and 50 ng of cDNA. The cycling parameters for RT-PCR were as follows: 95°C for 3 min, followed by 40 cycles of 95°C for 10 s, the required annealing temperature for 30 s, and 72°C for 40 s. Melting curve analysis was performed from 65°C, gradually increasing by 0.5°C/6 s to 95°C, to verify specificity. For each sample, RT-PCR reactions were completed in triplicate. Gene expression levels were quantified relative to the expression of β*-actin* using the optimized comparative Ct (2^–ΔΔCt^) value method (Dai et al., 2015). Data are presented herein as mean ± standard error of the mean (*n* = 3).

### Statistical Analysis

Statistical analyses were performed with SPSS version 19.0 software. Prior to conducting parametric analyses, all data were tested for normality using the Kolmogorov-Smirnov test and for homogeneity of variances using Levene’s test. Significant differences were evaluated by one-way analysis of variance (ANOVA), followed by Duncan’s multiple range tests. Differences were considered to be statistically significant when *P* < 0.05.

## Results

### Cloning and Bioinformatic Analysis of the *GHR* Gene

**FIGURE 1:**
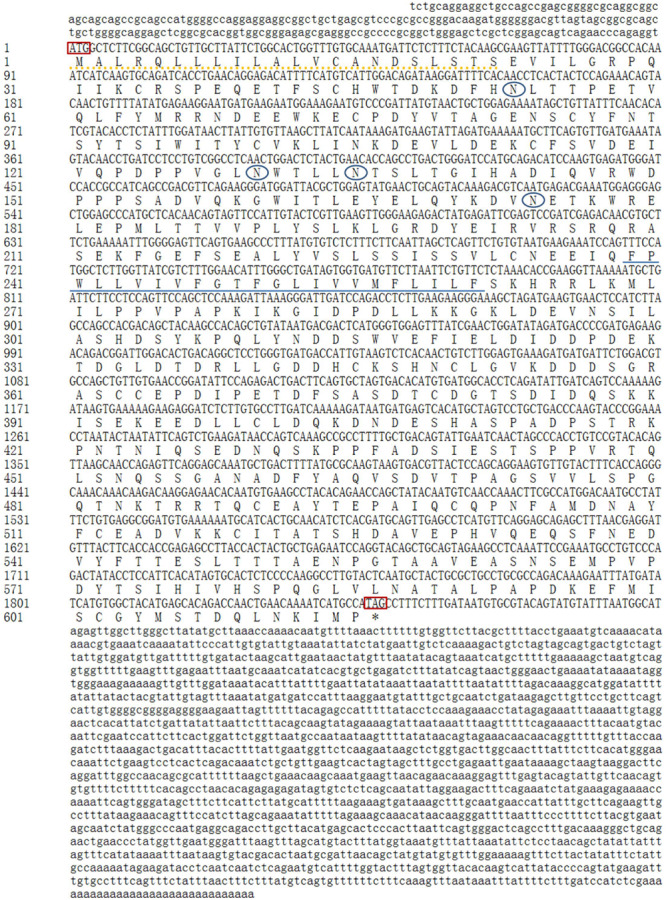
cDNA and deduced amino acid sequences of the *GHR* gene in Reeves’ turtle. The 5′-UTR and 3′-UTR are shown in lowercase, while the coding region is shown in uppercase. The upper sequence shows nucleotides, while the lower sequence shows amino acids. The initiation codon and the termination codon are indicated by a rectangular box. N-glycosylation sites are indicated by oval boxes. The putative signal peptide region is shown by a dotted line, and the potential transmembrane domain is underlined.

The full-length cDNA sequence for *GHR* in Reeves’ turtle (GenBank Accession Number: MH460865) was 3936 bp long and contained a 219 bp 5′UTR, a 1870 bp 3′UTR, and a 1848 bp ORF encoding 616 amino acids (Figure 1). A putative signal peptide of 22 amino acid residues was present at the N-terminal, and a potential transmembrane domain of 23 amino acids was located in the central region (239-261aa) of the GHR protein. Glycosylation site prediction suggested the existence of 4 N-glycosylation sites in the GHR protein (53, 130, 135, and 174aa).

**FIGURE 2:**
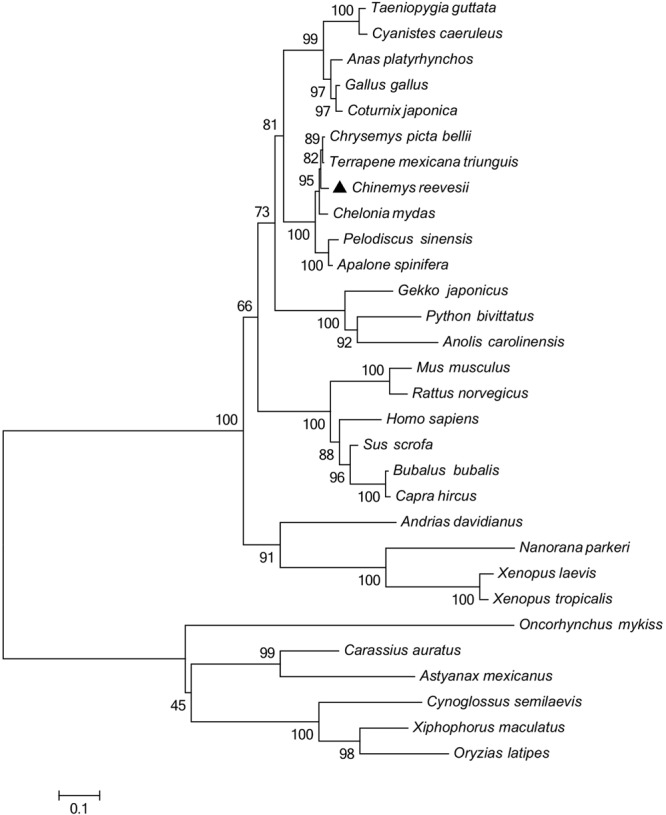
Phylogenetic analysis of GHR proteins. Only complete protein sequences were used for this analysis. The tree was constructed by the neighbor-joining method, using MEGA6.0 software (http://www.megasoftware.net/). The numbers at the nodes represent the bootstrap percentages from 1000 replicates. The GenBank accession numbers of GHR proteins used in the present study are listed in Table 3.

Multiple sequence alignments (Table 2) indicated that the *GHR* in Reeves’ turtle shared the highest amino acid sequence identities with Testudines (95.28–96.26%), followed by birds (70.77–75.91%), reptiles (64.25–65.08%), mammals (58.27–64.92%), amphibians (53.24–54.41%), and teleost fish (37.12–42.16%). These results were in accordance with phylogenetic analysis. As shown in Figure 2, the *GHR* in Reeve’s turtle was gathered with Testudines turtles and exhibited high levels of similarity with the *GHR* in birds. However, the *GHRs* in reptiles, mammals, amphibians, and teleost fish were clustered on other independent branches.

**TABLE 2:** Amino acid sequence identities of *GHR* in Reeves’ turtle compared with other vertebrates.

	**Cr**	**Cpb**	**Cm**	**Pb**	**Ac**	**Xl**	**Xt**	**Tg**	**Gg**	**Cj**	**Hs**	**Mm**	**Bb**	**Ca**	**Ol**
Cr	100.00														
Cpb	96.26	100.00													
Cm	95.28	95.93	100.00												
Pb	65.08	65.25	64.75	100.00											
Ac	64.25	64.58	64.58	74.14	100.00										
Xl	53.24	53.24	52.75	47.83	47.15	100.00									
Xt	54.41	53.91	53.58	47.99	48.15	92.70	100.00								
Tg	70.77	71.92	71.43	60.83	60.63	50.00	50.84	100.00							
Gg	75.91	76.63	76.24	62.62	60.93	50.42	51.43	82.40	100.00						
Cj	75.91	76.63	76.57	62.13	60.93	50.08	51.10	82.89	97.53	100.00					
Hs	64.92	65.25	64.59	56.09	54.23	46.84	47.84	59.90	61.03	61.36	100.00				
Mm	58.27	58.59	57.77	53.44	52.11	46.68	46.84	56.34	57.95	57.78	68.81	100.00			
Bb	62.99	63.65	63.32	54.46	54.41	47.83	48.33	59.93	60.57	60.40	77.29	71.14	100.00		
Ca	42.16	42.16	41.80	40.32	37.90	38.25	38.07	40.65	40.69	40.33	40.53	39.19	40.85	100.00	
Ol	37.12	36.94	36.76	34.52	35.29	34.24	34.43	36.84	36.68	36.13	34.45	34.90	35.98	49.74	100.00

**TABLE 3:** Species names, common names, and GenBank numbers.

**Species**	**Common name**	**GenBank number**
*Chinemys reevesii*	Reeves’ turtle	MH460865
*Chrysemys picta bellii*	Western painted turtle	XP_023961406.1
*Pelodiscus sinensis*	Chinese soft-shelled turtle	XP_006139558.1
*Chelonia mydas*	Green sea turtle	XP_007052892.1
*Apalone spinifera*	Spiny softshell turtle	ATP07247.1
*Terrapene mexicana triunguis*	Three-toed box turtle	XP_024058900.1
*Python bivittatus*	Burmese python	XP_007426794.1
*Anolis carolinensis*	green anole	XP_016846368.1
*Gekko japonicus*	Japanese Gecko	XP_015279964.1
*Xenopus laevis*	African clawed frog	AAF05775.1
*Xenopus tropicalis*	Tropical clawed frog	XP_002933093.2
*Nanorana parkeri*	Tibetan Plateau frog	XP_018417806.1
*Andrias davidianus*	Chinese giant salamander	ALD83575.1
*Taeniopygia guttata*	Zebra Finc	XP_002193695.2
*Cyanistes caeruleus*	Blue tit	XP_023800353.1
*Anas platyrhynchos*	Mallard	NP_001297276.1
*Gallus*	Chicken	AGG38007.1
*Coturnix japonica*	Japanese quail	XP_015704162.1
*Homo sapiens*	Human	EAW56023.1
*Mus musculus*	House mouse	AAH75720.1
*Bubalus bubalis*	Water buffalo	ABM92306.2
*Sus scrofa*	Pig	ABD77501.1
*Rattus norvegicus*	Norway rat	NP_058790.1
*Capra hircus*	Goat	ABS11661.1
*Carassius auratus*	Goldfish	AAK60495.1
*Cynoglossus semilaevis*	Tongue sole	NP_001281126.1
*Oncorhynchus mykiss*	Rainbow trout	NP_001118203.1
*Astyanax mexicanus*	Mexican tetra	XP_022526167.1
*Xiphophorus maculatus*	Southern platyfish	XP_023199584.1
*Oryzias latipes*	Japanese medaka	XP_011477148.1

### The Tissue Distribution of *GHR* and *IGF-I* mRNA in Reeves’ Turtle

**FIGURE 3:**
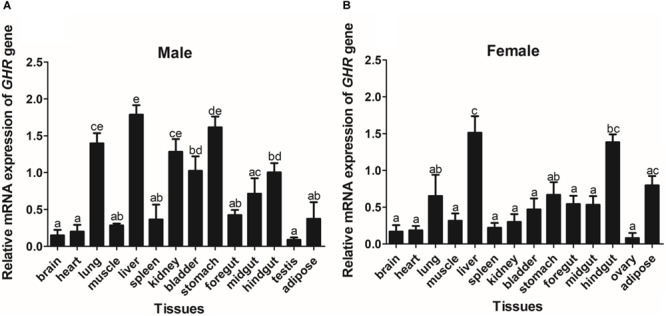
The tissue distribution of the *GHR* gene in male **(A)** and female **(B)** Reeves’ turtle. Gene expression levels were evaluated by the comparative Ct (2^–△△Ct^) method with β*-actin* as an internal standard. Significant differences between groups were detected using one-way ANOVA. Different superscripts signify significant differences (*P* < 0.05). Data are expressed as mean ± standard error of the mean (*n* = 3).

**FIGURE 4:**
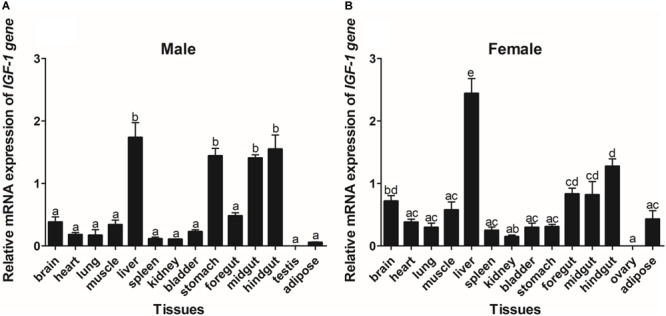
Tissue distribution of the *IGF-I* gene in male **(A)** and female **(B)** Reeves’ turtles. Gene expression levels were evaluated by the comparative Ct (2^–△△Ct^) method with β*-actin* as an internal standard. Significant differences between groups were detected using one-way ANOVA. Different superscripts signify significant differences (*P* < 0.05). Data are expressed as mean ± standard error of the mean (*n* = 3).

The expression of *GHR* and *IGF-I* mRNA was detected in various tissue samples harvested from Reeves’ turtle, using RT-PCR. Figures 3, 4 show that *GHR* and *IGF-I* mRNAs were widely expressed, and that there were differences in expression between the two sexes.

In male turtles, the *GHR* gene was mainly expressed in the liver, stomach, lung, and kidney, with moderate expression in the muscle, spleen, bladder, gut, and adipose tissue. Expression levels were low in the brain, heart, and testis (Figure 3A). The mRNA expression levels of *IGF-I* were significantly higher in the liver, stomach, midgut, and hindgut tissue than in the brain, heart, lung, muscle, spleen, kidney, bladder, foregut, testis, and adipose tissues (*P* < 0.05) (Figure 4A). However, in females, high *GHR* expression levels were observed in the liver, hindgut, adipose tissue, stomach, and lung (Figure 3B), and in the liver, gut and brain for *IGF-I* (*P* < 0.05) (Figure 4B). Notably, both *GHR* and *IGF-I* showed the highest mRNA expression levels in the liver, and the lowest levels in gonadal tissue (*P* < 0.05); this was the case for both males and females.

### The Effect of Fasting and Refeeding on *GHR* Expression

**FIGURE 5:**
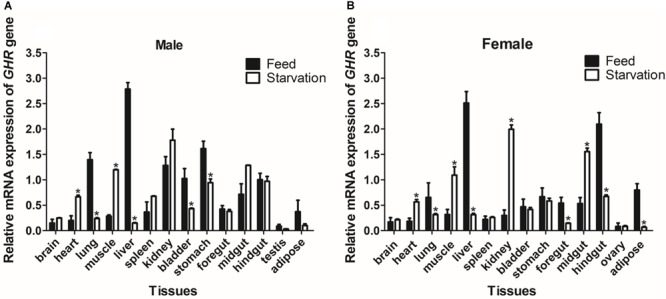
The expression of *GHR* mRNA in male **(A)** and female **(B)** Reeves’ turtles in a group that was fed and a group that was starved for 28 days. Expression levels were normalized against those of β*-actin* as an internal standard. Groups that differed significantly are indicated by asterisks above the bars (*P* < 0.05). Data are shown as mean ± standard error of the mean (*n* = 3).

To determine the effect of fasting on *GHR* mRNA expression, we detected mRNA levels in a variety of different tissues and compared these levels between the group of turtles that was fed and the group that was starved. After 28 days of fasting, the levels of *GHR* mRNA expression were significantly increased in the heart and muscle, but significantly decreased in the lung, liver, bladder, and stomach tissues (*P* < 0.05) in males (Figure 5A). In females, the expression levels of *GHR* in the starvation group were significantly increased in the heart, muscle, kidney, and midgut when compared with the group of turtles that were fed, but were significantly decreased in the lung, liver, foregut, hindgut and adipose tissues (*P* < 0.05) (Figure 5B).

**FIGURE 6:**
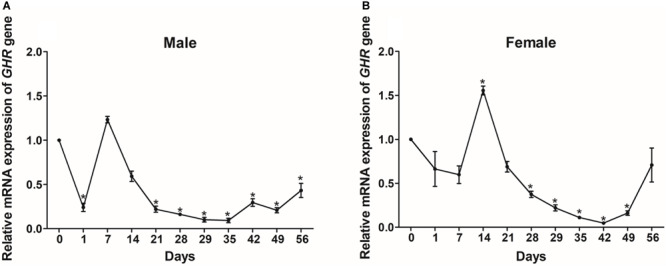
The relative gene expression of *GHR* in the livers of male **(A)** and female **(B)** Reeves’ turtle during fasting (day 0–28) and refeeding (day 28–56). Following standardization against β*-actin*, gene expression levels were normalized to an expression level of 1 on day 0 (baseline). Asterisks above the bars indicate significant differences (*P* < 0.05). Data are shown as mean ± standard error of the mean (*n* = 3).

We also monitored the changes in *GHR* expression in the livers of Reeves’ turtles during the fasting and refeeding process. As shown in Figure 6A, *GHR* expression in male turtles decreased significantly (*P* < 0.05) during the fasting process, with a transient increase between days 1 and 7. During the refeeding process, *GHR* transcript levels were relatively stable between days 28 and 56 during which expression was significantly lower (*P* < 0.05) than baseline (day 0). In females, the mRNA expression levels of *GHR* increased initially during the fasting period and then decreased; expression levels were significantly increased on day 14 and significantly decreased on day 28 (*P* < 0.05). During the refeeding period, the expression levels of *GHR* declined continuously until day 42; levels then increased and had reached the normal baseline level by day 56 (Figure 6B).

### The Effect of Fasting and Refeeding on the Expression of the *IGF-I* Gene

**FIGURE 7:**
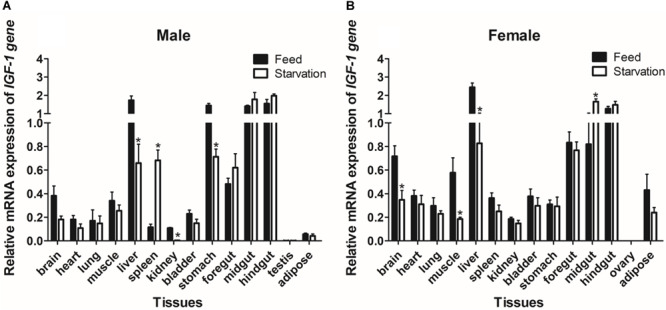
The expression of *IGF-I* mRNA in male **(A)** and female **(B)** Reeves’ turtle in groups that were fed and starved for 28 days. Expression levels were normalized against β*-actin* as a reference gene. Groups that differed significantly are indicated by asterisks above the bars (*P* < 0.05). Data are shown as mean ± standard error of the mean (*n* = 3).

We also evaluated the levels of *IGF-I* mRNA expression in groups of Reeves’ turtles in group that was fed and a group that was not fed in order to study the effect of starvation on gene expression. Our analysis showed that the expression of *IGF-I* mRNA in males from the starvation group was significantly down-regulated (*P* < 0.05) in the liver, kidney, and stomach tissues, while the expression of *IGF-I* mRNA in the spleen was significantly upregulated (*P* < 0.05) (Figure 7A). In comparison with turtles that were fed, female turtles from the starvation group exhibited a significant upregulation of *IGF-I* mRNA expression in the brain, muscle, and liver (*P* < 0.05); in contrast, *IGF-I* mRNA was significantly down-regulated (*P* < 0.05) (Figure 7B).

**FIGURE 8:**
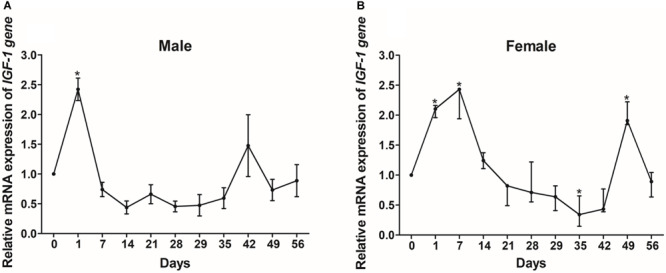
The relative gene expression of *IGF-I* in the livers of male **(A)** and female **(B)** Reeves’ turtle during fasting (days 0–28) and refeeding (days 28–56). Following standardization by β*-actin*, gene expression levels were normalized to a mean expression level of 1 on day 0 (baseline). Asterisks above the bars indicate significant differences (*P* < 0.05). Data are shown as mean ± standard error of the mean (*n* = 3).

In order to investigate how nutritional status regulates *IGF-I*, we investigated the mRNA expression levels of *IGF-I* in the livers of Reeves’ turtles under different feeding regimes. In males, the expression levels of *IGF-I* showed a significant increase after just one day (*P* < 0.05) but did not show any significant changes thereafter for the rest of the fasting and refeeding process (Figure 8A). *IGF-I* expression in female turtles first showed a significant increase between days 1 and 7, and then decreased during the fasting process (Figure 8B). However, *IGF-I* mRNA expression showed the opposite tendency during the refeeding process, in that expression levels decreased significantly on day 35 and increased significantly on day 49 (*P* < 0.05).

## Discussion

The regulation of growth has become a significant area of endocrine research over recent years. The GH/IGF axis plays an important role in coordinating the growth of vertebrates (Reindl and Sheridan, 2012). As a core member of the GH/IGF axis, the GHR has received particular attention from researchers, particularly with regards to identification and functional expression. The *GHR* gene was initially discovered in mammals (Leung et al., 1987). Subsequently, the *GHR* cDNA sequence was identified in a range of other species (Fukada et al., 2004; Zhong et al., 2012; Botta et al., 2016). However, until now, little was known about the *GHR* gene in Testudoformes. In the present study, we cloned and characterized the full-length cDNA sequence of *GHR* for the first time in Reeves’ turtle. According to the deduced amino acid sequence, the GHR protein was composed of 616 amino acids, including a putative signal peptide of 22 amino acids, an extracellular domain of 216 amino acids, a single transmembrane domain of 23 amino acids, and intracellular domain of 356 amino acids. Four potential N-glycosylation sites were located in the extracellular domain of GHR; these sites are highly conserved across different species and have been reported to be important for ligand binding and physiological activity (Zhang et al., 2000). Phylogenetic analysis and multiple alignments of the GHR protein revealed that the *GHR* in Reeves’ turtle showed the highest levels of sequence identity with birds (70.77–75.91%), followed by reptiles (64.25–65.08%), mammals (58.27–64.92%), amphibians (53.24–54.41%), and teleost fish (37.12–42.16%). These results were consistent with those from previous research studies, and support the fact that the Reeves’ turtle is a sister group of birds and crocodiles (Chiari et al., 2012; Wang et al., 2013; Crawford et al., 2015).

The expression patterns of *GHR* in a range of tissue samples from Reeves’ turtle indicated that the *GHR* gene was universally expressed; this finding was consistent with research carried out in other vertebrates (Zeng et al., 2014). In Reeves’ turtle, *GHR* and *IGF-I* exhibited the highest expression levels in the liver, thus indicating that this organ was the target organ of GH. Furthermore, tissue distribution analysis indicated that *GHR* and *IGF-I* were expressed widely, suggesting that these genes play a role in an extensive range of biological functions, including metabolism, gonadal development, osmoregulation, social behavior, and immunity (Bergan-Roller and Sheridan, 2018). Notably, we observed higher levels of *IGF-I* expression in the liver tissues of males than in those of females. These observations might indicate that male and female development is not synchronous during the early stages of body growth, and that the expression of key genes involved in the GH/IGF*-I* axis may play a role in sex differences (Ma et al., 2016). In the present study, we were unable to detect *IGF-I* in either male or female gonadal tissue. These results suggest a synergistic role for IGFs during the development of gonadal tissue in Reeves’ turtle. Similar mechanisms have been proposed for other vertebrates (Armstrong et al., 2000).

Reduced nutritional status inevitably causes changes in the secretion of hormones in animals and thus alters metabolic levels to facilitate survival. During this process, animals transfer energy for growth and storage to maintain physiological basal metabolism. It is well-known that Reeves’ turtle can survive for long periods of time without feeding. However, prior to this study, little was known about the changes that occur in the expression patterns of *GHR* and *IGF-I* change in this species when subjected to food deprivation. The somatotropic axis is a neuroendocrine system consisting of a series of hormones and receptors extending from the hypothalamus to the pituitary, and to various target organs (Gomez-Requeni et al., 2005). Within the somatotropic axis, the GH/IGF-I axis plays a critical role in the process of animal growth (Gray and Kelley, 1991). GH is first released by the pituitary gland and then travels to the liver *via* the blood circulation; it then binds to GHRs on the surface of the liver cell membrane, activates JAK2, and induces the expression of *IGF-I*. The IGF-I protein is transported to its target tissue by the circulatory system, and exerts a series of biological effects, including increased glucose uptake and the inhibition of fat and protein breakdown (Pierce et al., 2004). In the present study, we found that the levels of *GHR* mRNA in the livers of male or female Reeves’ turtle were significantly reduced after 28 days of fasting. This indicated that the expression of the *GHR* gene in this species is inhibited when nutrient levels are reduced. A previous study, involving rats, reported that the expression levels of *GHR* in the liver also decreased significantly during fasting (Ohashi et al., 1995). In a study of porcine liver cells, Brameld et al. (1999) found that the expression levels of *GHR* mRNA in a group of cells with reduced glucose content decreased by almost 40%; in addition, the arginine and glycine content of these cells also decreased. The expression of *GHR* mRNA decreased only slightly in a group of cells with normal glucose levels (Brameld et al., 1999). We believe that nutritional status is a key regulator of the expression of *GHR* in the liver of Reeves’ turtles. Future studies should aim to determine which specific nutrients affect the expression of GHR in this species.

On the first day of refeeding, the expression levels of *GHR* mRNA in the liver of Reeves’ turtles were significantly lower that at baseline. By the fourth week of refeeding, the expression levels of *GHR* mRNA in livers from males were still significantly lower than pre-fasting expression levels. This may be related to the lower food intake and metabolic levels of Reeves’ turtle (Polakof et al., 2011; Liu W.S. et al., 2016). The expression levels of *GHR* mRNA in females decreased continuously during the 2 weeks of refeeding. However, by week 4 of refeeding, the expression levels of *GHR* mRNA in females were not significantly different to those seen prior to fasting. Interestingly, throughout refeeding, the expression levels of *GHR* mRNA in the livers of males were significantly lower than the pre-fasting expression levels. This phenomenon implies that the endocrine response in males occurs faster under starvation stress than in females, although males are less resilient than females. We speculate that this effect may be related to sexual dimorphism (Lu et al., 2015), because similar findings have also been reported for *Pelteobagrus fulvidraco* (Ma et al., 2016; Zhang et al., 2016), the mouse (Gesing et al., 2015; Liu Z. et al., 2016), and *Oreochromis hornornum* (Gao et al., 2011).

As an important growth factor in the GH/IGF axis, IGF-I has a range of physiological functions, including the regulation of cell metabolism, the promotion of cell proliferation and differentiation, the inhibition of apoptosis, and the regulation of reproductive and immune-related hormonal secretion (Sara and Hall, 1990). Following its production in the liver, IGF-I is released into the blood and travels to its target tissue via the blood (Epa and Ward, 2006). Studies have shown that the levels of *IGF-I* mRNA in the livers of fish are significantly reduced under starvation (Yi, 2001; Chen et al., 2010). After 28 days of food deprivation, we also found a significant reduction of *IGF-I* mRNA in the liver of Reeves’ turtle. Other studies have also reported a reduction in the expression of hepatic IGF-I in the salmon and eel after 2–4 weeks of fasting (Duan and Hirano, 1992; Duan and Plisetskaya, 1993). Another study reported a reduction in the levels of *IGF-I* mRNA in the livers of rats when starved for 3 days (Bornfeldt et al., 1989). A significant reduction of *IGF-I* mRNA in the liver was also observed in cockerels after fasting for 5 days (Morishita et al., 1993). In the present study, we observe a significant reduction in the levels of *IGF-I* mRNA in the livers of Reeves’ turtles after 28 days of fasting; these findings were consistent with studies involving other poikilotherms (Strobel et al., 2020). It is likely that these results are due to the low metabolic rates in both fish and Reeves’ turtles. Besides, the expression trend of *IGF-I* mRNA is inconsistent with that of *GHR* mRNA in both male and female in different nutritional state. The growth hormone released by the pituitary gland reaches the liver through blood circulation, and then binds to the growth hormone receptor on the surface of the liver cell membrane. After that, the expression of *IGF-I* gene was activated, but maybe there is a certain lag in this regulation.

During the early stages of recovery feeding, we found that the mRNA levels of *IGF-I* in female Reeves’ turtle were still significantly reduced; similar results were observed in a study involving rainbow trout (Wilkinson et al., 2006). It is evident that nutritional status can regulate the transcription of *IGF-I*, although there appears to be a lag period in this form of regulation. During the first week of refeeding, the expression levels of *IGF-I* in the livers of males increased to the levels seen before fasting. However, after 3 weeks of refeeding, the expression levels of *IGF-I* in the livers of females were significantly higher than those measured before fasting. Other studies have shown that fish can exhibit compensatory growth; after 4 weeks of fasting, *Pelodiscus sinensis* showed a significantly higher specific growth rate than a control group during the first week of refeeding (Xie and Niu, 2007). We speculate that the rapid rise of *IGF-I* in Reeves’ turtle may be related to compensatory growth owing to its critical role in promoting growth and development. In view of the critical role of GHR, we believe that the effect of turtle GHR gene on cell proliferation, differentiation and apoptosis should be further studied.

## Conclusion

In this study, we cloned the full-length cDNA for the *GHR* gene in Reeves’ turtle for the first time. Compared with mammals, the expression patterns of *GHR* and *IGF-I* mRNA in this species of turtle are more similar to those of poikilotherms, including fish. We also demonstrated that external nutritional conditions can induce significant changes in the mRNA levels of *GHR* and *IGF-I* in the liver of the Reeves’ turtle. There were significant differences in the expression levels of *GHR* and *IGF-I* when compared between the gut, muscle, and kidney during periods of food deprivation. These findings provide a useful foundation from which to further investigate the potential mechanisms involved in the ability of Reeves’ turtle to endure starvation.

## Data Availability Statement

The datasets generated for this study can be found in the Genebank accession number: MH460865.

## Ethics Statement

The animal study was reviewed and approved by the Animal Care Committee of South China Agriculture University (Guangzhou, China). Written informed consent was obtained from the owners for the participation of their animals in this study.

## Author Contributions

WZ, YH, and ZR conceived the study, designed the project, and helped draft the manuscript. LL and YG analyzed the sequencing data. NL, RL, and XC performed qRT-PCR. XZ provided advice relating to the research. WL helped to draft the manuscript. All authors read and approved the manuscript.

## Conflict of Interest

The authors declare that the research was conducted in the absence of any commercial or financial relationships that could be construed as a potential conflict of interest.

## Footnotes

1 https://blast.ncbi.nlm.nih.gov/Blast.cgi

2 https://www.ncbi.nlm.nih.gov/

3 https://www.ncbi.nlm.nih.gov/orffinder/

4 http://www.cbs.dtu.dk/services/SignalP/

5 http://www.cbs.dtu.dk/services/NetNGlyc/

6 http://www.cbs.dtu.dk/services/TMHMM/

7 http://www.ebi.ac.uk/Tools/msa/clustalw2/

8 http://www.megasoftware.net/
